# Weekly versus triweekly cisplatin-based concurrent chemoradiotherapy in the treatment of locally advanced cervical carcinoma: An updated meta-analysis based on randomized controlled trials

**DOI:** 10.1097/MD.0000000000018663

**Published:** 2020-01-03

**Authors:** Jiahao Zhu, Zheng Zhang, Dongyan Bian, Qingqing Chen, Qunchao Hu, Shengjun Ji, Ke Gu

**Affiliations:** a Department of Radiotherapy and Oncology, Affiliated Hospital of Jiangnan University, Wuxi; b Department of Radiotherapy and Oncology, Suzhou Ninth People's Hospital, Suzhou; c Jiangsu Key Laboratory of Immunity and Metabolism, Department of Pathogenic Biology and Immunology, Xuzhou Medical University; d Department of Radiotherapy and Oncology, The Affiliated Suzhou Hospital of Nanjing Medical University, Suzhou, Jiangsu, P.R. China.

**Keywords:** chemoradiotherapy, cisplatin, locally advanced cervical carcinoma, triweekly, weekly

## Abstract

**Background:**

Radiotherapy concurrent with cisplatin is the standard regimen used for treatment of locally advanced cervical carcinoma. In this meta-analysis, survival, recurrence, compliance, and acute adverse effects were compared between weekly and triweekly cisplatin-based concurrent chemoradiotherapy regimens for treatment of cervical cancer.

**Methods:**

A systematic search for relevant studies was conducted using PubMed, Cochrane Library, EMBASE, and Medline databases. Fixed- or random-effects models were used for pooled analysis. The endpoints were overall survival, recurrence, compliance, and acute adverse effects reported as odds ratios (ORs) and 95% confidence intervals (CIs).

**Results:**

Eight randomized controlled trials met the inclusion criteria. No significant differences were observed between the 2 arms with respect to recurrence, survival, and acute adverse effects (all *P* > .05). However, the triweekly cisplatin regimen was associated with significantly lower incidence of local recurrence (OR, 1.72; 95% CI, 1.07–2.78; *P* = .03), radiotherapy completion (OR, 2.08; 95% CI, 0.99–4.38; *P* = .05), and anemia (OR, 2.10; 95% CI, 1.01–4.37; *P* = .05), while a weekly cisplatin regimen was associated with a lower risk of leukopenia (OR, 0.57; 95% CI, 0.42–0.92; *P* = .00) and thrombocytopenia (OR, 0.55; 95% CI, 0.31–0.97; *P* = .04).

**Conclusions:**

Triweekly cisplatin-based chemotherapy significantly reduced local recurrence with tolerable toxicity and might be the optimal regimen in concurrent chemoradiotherapy for locally advanced cervical carcinoma.

## Abstract 2

Supplemental Digital Content is available in the text

## 1 Introduction

Cervical carcinoma is one of the most common malignancies in women worldwide, and locally advanced stages of the disease account for both the majority of patients diagnosed and cervical carcinoma-associated deaths.^[1]^ Different treatments are adopted based on the International Federation of Obstetrics and Gynecology stage. For locoregionally advanced disease (stages IIB, III, and IVA), cisplatin-based concomitant chemoradiotherapy (RT) has been the primary treatment modality recommended by the US National Cancer Institute since 1992, based on 5 randomized clinical trials.^[2–6]^ Despite trials addressing different chemotherapy regimens, and the improvements on RT technology and equipment, there is still a significant risk of both recurrence and poor prognosis in patients with locally advanced cervical cancer (LACC).^[7]^

Weekly and triweekly single cisplatin dosing schedules concurrent with RT are commonly adopted for treatment of LACC. Although cisplatin-based doublet chemotherapy regimen shows better overall survival (OS) and progression-free survival (PFS), adverse reactions are also significantly increased.^[8]^ Among the 5 abovementioned randomized clinical trials, 2 trials studied weekly cisplatin administration, while the other 3 used a triweekly cisplatin regimen. However, the optimal chemotherapy regimen is yet to be established. Hu et al subsequently conducted a meta-analysis to evaluate the efficacy of weekly and triweekly cisplatin combined with RT for the treatment of cervical cancer. They found that weekly cisplatin was associated with a lower risk of hematological toxicity than triweekly cisplatin with concurrent chemoradiotherapy (CCRT). However, the 2 regimens were comparable regarding PFS and OS (*P* > .05),^[9]^ which is similar to the results of another meta-analysis conducted by Chen et al.^[10]^ However, both of the meta-analyses included a small number of randomized controlled trials. In addition, 2 duplicate articles and 1 retrospective study were included in the meta-analysis of Hu et al. Therefore, 8 randomized trials were included in our meta-analysis to explore the differences between the cisplatin regimens for patients with LACC.

## 2 Materials and methods

### 2.1 Search strategy

PubMed, Cochrane Library, EMBASE, and Medline databases were searched for relevant articles using the following Keywords: (Cisplatin OR Platinum OR cis-Platinum OR Platinol OR Platidiam OR CDDP), (Uterine Cervical Neoplasms OR Cervical Neoplasms OR Cervix Neoplasms OR Uterine Cervix Cancers OR Cervix Cancers OR Cervical Cancers), (Triweekly OR Every three weeks OR Three weeks), (Per week OR Every week OR Weekly OR Once a week), AND (chemoradiotherapy OR chemoradiation OR radiochemotherapy OR chemotherapy OR radiotherapy OR radiation OR electromagnetic radiation). Only studies published in English from January 1, 1990 to December 29, 2017 were considered. The references of the included studies and related citations were also checked manually for potentially relevant studies. Two independent investigators evaluated each study. A consensus was reached by discussion or by consulting a third investigator to resolve the disagreements between the 2 reviewers.

### 2.2 Inclusion and exclusion criteria

Studies were included in the analysis if:

(1) they were randomized controlled trials that compared triweekly cisplatin plus RT versus weekly single cisplatin plus RT;
(2) there was no evidence of para-aortic lymph nodes or distant metastasis on pretreatment imaging (stages I to IVA); and
(3) the long-term OS and recurrence rate including local and distant were assessed as outcomes to measure the effect of the treatment.

If studies were duplicates, the study with the most up-to-date results was included. Studies were excluded if patients had previous history of chemotherapy or RT or other factors seriously affecting the survival and treatment processes.

We use the revised Jadad scale to evaluate the quality of the randomized controlled trials included in the primary outcome analysis. Articles with a high quality scored 4 to 7 points. The Ethical Committee of Suzhou Municipal Hospital approved this study.

### 2.3 Statistical analysis

OS and recurrence rate, including the locoregional relapse rate and rate of distant metastasis, were the primary endpoints, and appliance and acute adverse reactions were the secondary endpoints. RevMan 5.3 software (Cochrane Collaboration's Information Management System) was used to conduct this meta-analysis. Variables among studies with minimal heterogeneity were assessed by a fixed-effect model/Mantel–Haenszel method and, otherwise, a random-effects model/DerSimonian–Laird method was used when calculating the odds ratios (ORs) and 95% confidence intervals (CIs) for each specific event. *I*^2^ < 60% was defined as low heterogeneity and a fixed-effects model was used in our study; otherwise, a random-effects model was adopted. Funnel plots and Harbord tests were used to examine potential publication bias in the meta-analysis.

## 3 Results

### 3.1 Study selection and characteristics

**Figure 1:**
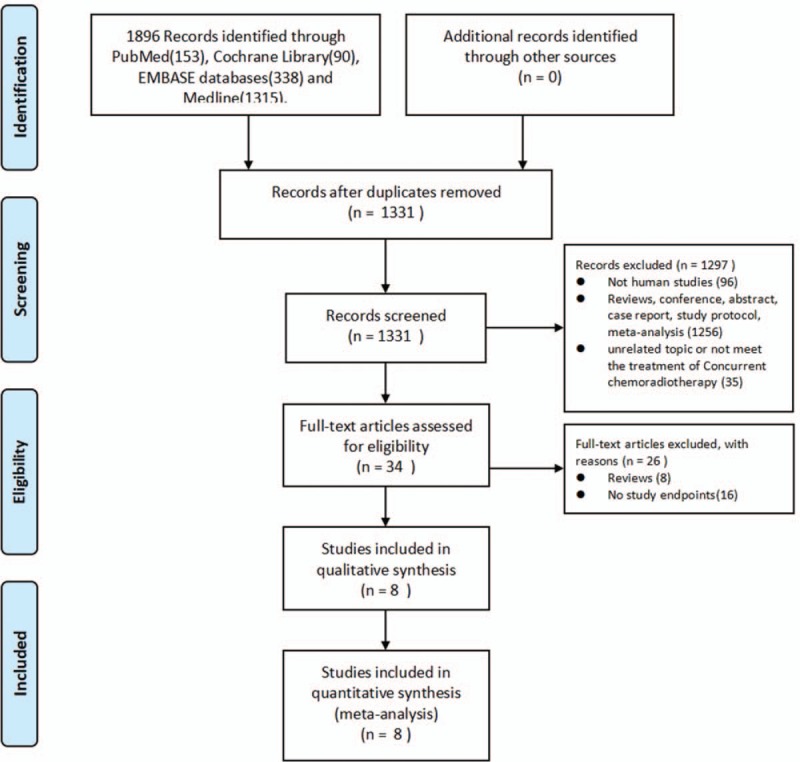
Flow diagram outlining the identification of retrieved publications.

The search initially yielded a total of 1896 citations. Eight^[11–18]^ of these were included in this review after excluding studies that did not meet the inclusion criteria or were duplicate publications, review articles, or meta-analyses. Two trials were not included due to a lack of availability of relevant data. The study selection criteria for this meta-analysis are presented in Figure 1.

All 8 publications considered in this analysis were prospective randomized trials. The 8 studies, with a combined sample size of 1176 patients, were conducted in the US, Japan, India, Korea, and Romania and were published between 2007 and 2017. All of the patients recruited in these studies were newly diagnosed as LACC and received primary radical CCRT. Out of the 1176 patients, 587 received weekly cisplatin-based CCRT, while 338 patients received a triweekly regimen, and 251 patients received the treatment every 4 weeks. Table 1 shows a detailed analysis of the studies.

**Table 1:**
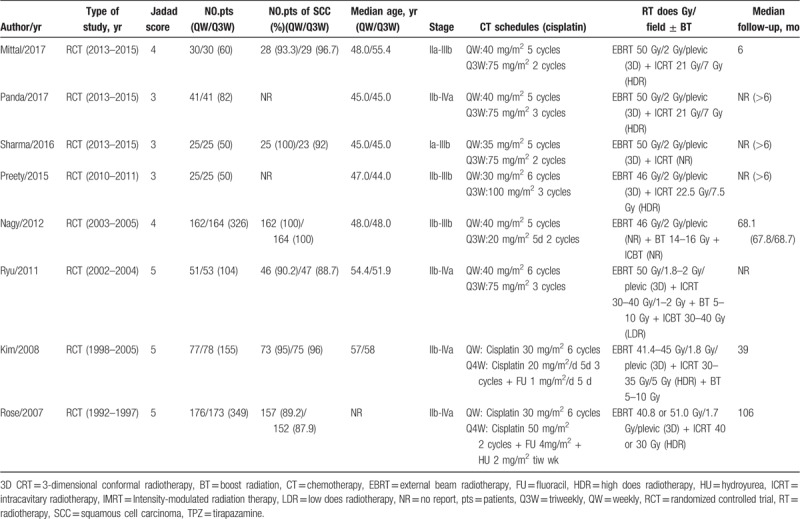
Characteristics of the included trials.

### 3.2 Primary endpoints: OS and recurrence rate

**Figure 2:**
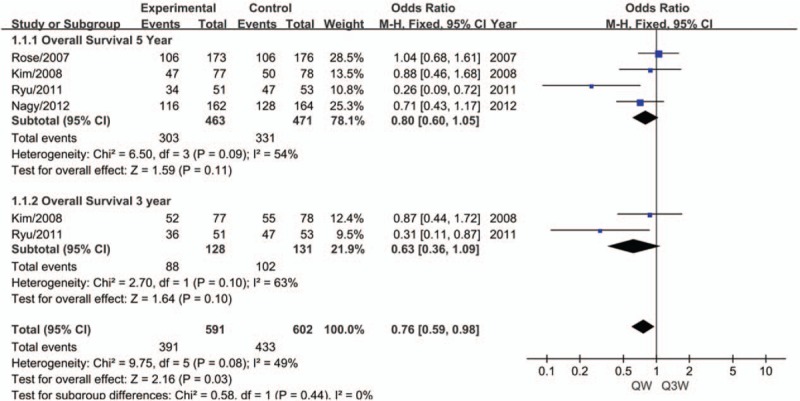
Meta-analysis evaluating the overall survival of weekly or triweekly cisplatin combined with radiotherapy.

**Figure 3:**
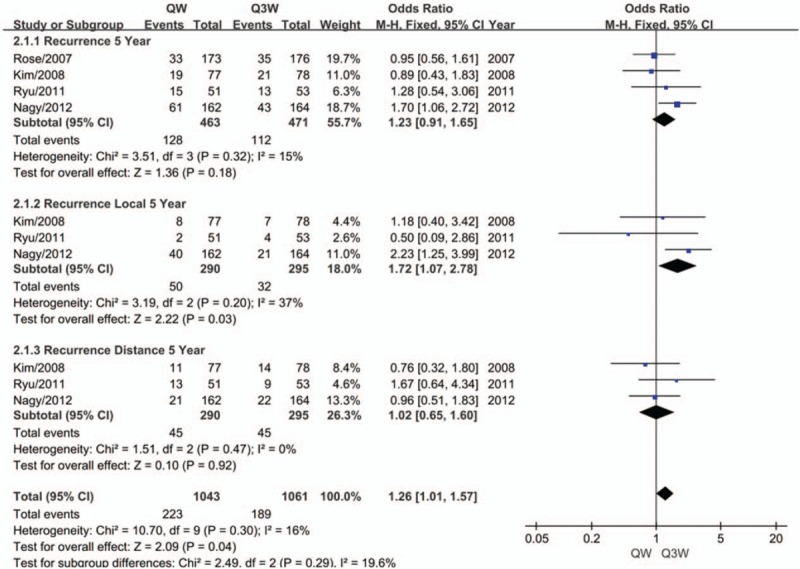
Meta-analysis evaluating the recurrence of weekly or triweekly cisplatin combined with radiotherapy.

The meta-analysis of 5-year OS (n = 4 studies) and 3-year OS (n = 2 studies) showed no significant heterogeneity among these trials. Therefore, the fixed-effects model was chosen for pooled analysis. Both Harbord tests showed a lack of significant heterogeneity among the trials (*P* > .05). The analysis revealed no statistically significant difference between the triweekly regimen or the weekly regimen of the cisplatin-based CCRT compared to 5-year OS (OR, 0.80; 95% CI, 0.60–1.05; *P* = .11; Fig. 2) and 3-year OS (OR, 0.63; 95% CI, 0.36–1.09; *P* = .10; Fig. 2).

No significant heterogeneity was observed among the trials for the meta-analysis of the recurrence rate (n = 4 studies). Therefore, the fixed-effects model was chosen for pooled analysis. No significant difference was found between the 2 regimens of CCRT with respect to 5-year recurrence (OR, 1.23; 95% CI, 0.91–1.65; *P* = .18; Fig. 3). We performed subgroup analysis in terms of 5-year recurrence and found that triweekly cisplatin plus RT was associated with a 37.1% reduced risk of 5-year local recurrence compared to weekly cisplatin-based CCRT (OR, 1.72; 95% CI, 1.07–2.78; *P* = .03; Fig. 3). However, no significant difference was observed between the 2 regimens of CCRT with respect to 5-year distant recurrence (OR, 1.02; 95% CI, 0.65–1.60; *P* = .92; Fig. 3).

### 3.3 Secondary endpoints: compliance and acute adverse events

**Figure 4:**
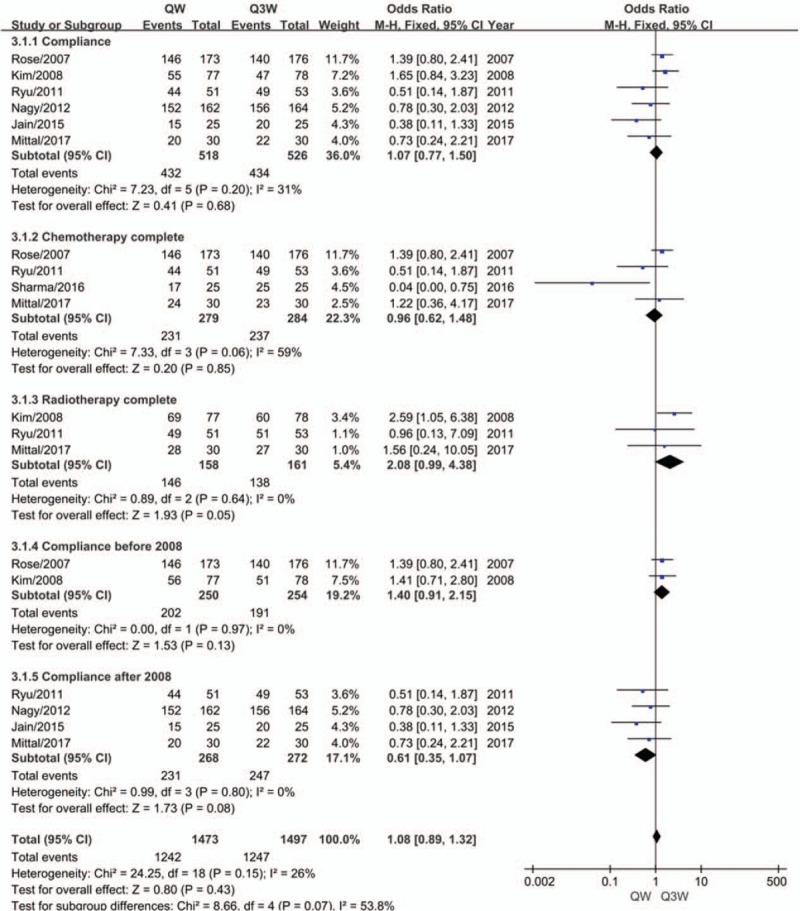
Meta-analysis evaluating the compliance with weekly or triweekly cisplatin combined with radiotherapy.

Patients with poor compliance were defined as those not completing the total number of cycles of chemotherapy, missing a dose of cisplatin, having a delayed radiation period longer than a certain period of time, and having other variations. For the meta-analysis of compliance (n = 6 studies), no significant heterogeneity was observed among the trials. Therefore, the fixed-effects model was chosen for pooled analysis. No significant difference was shown between the 2 regimens of CCRT with respect to compliance (OR, 1.07; 95% CI, 0.77–1.50; *P* = .68; Fig. 4). We performed subgroup analysis in terms of compliance and found that patients treated with triweekly cisplatin plus RT had a lower rate of completed RT (OR, 2.08; 95% CI, 0.99–4.38; *P* = .05; Fig. 4). Moreover, an interesting phenomenon was observed as a strong trend of better compliance took place in patients treated with triweekly cisplatin plus RT after 2008 (OR, 0.61; 95% CI, 0.35–1.07; *P* = .08; Fig. 4).

**Figure 5:**
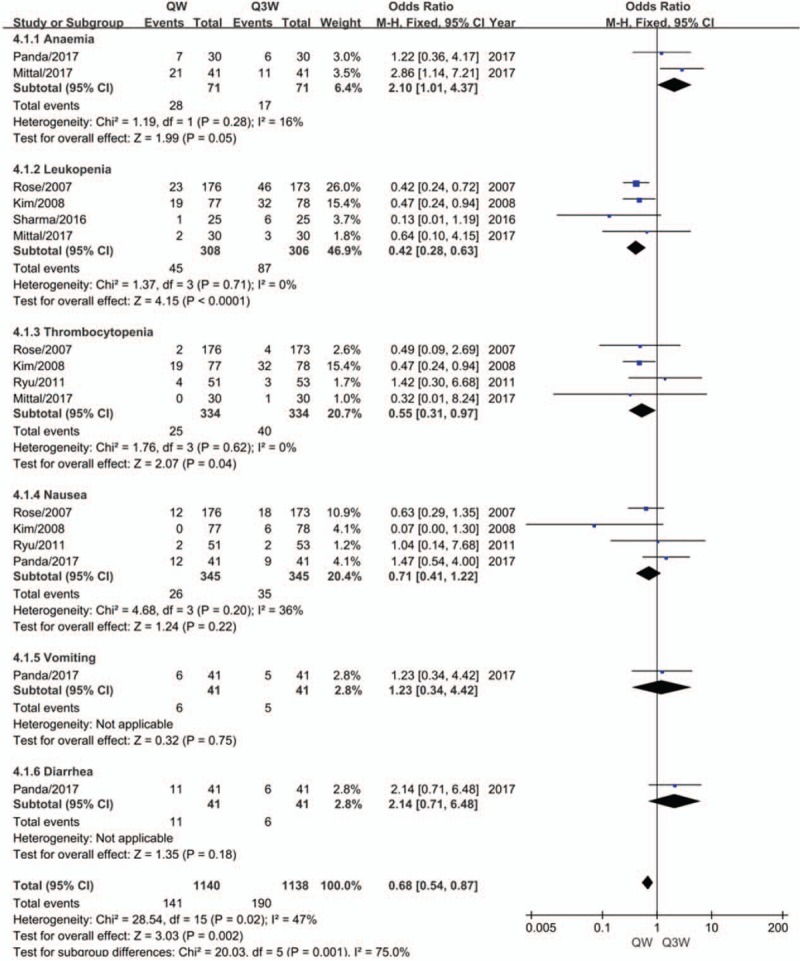
Meta-analysis evaluating the hematological and gastrointestinal toxicity of weekly or triweekly cisplatin combined with radiotherapy.

We compared grade 3 or 4 anemia, leukopenia, and thrombocytopenia as indicators of hematological toxicity between the 2 regimens. Grade 3 or 4 nausea, vomiting, and diarrhea were analyzed for gastrointestinal acute reactions. We observed that the triweekly group suffered less from anemia (OR, 2.10; 95% CI, 1.01–4.37; *P* = .03; Fig. 5) but doubled the incidence rate of leukopenia (OR, 0.42; 95% CI, 0.28–0.63; *P* = .00; Fig. 5), and presented an increased incidence rate of thrombocytopenia by 60% (OR, 0.55; 95% CI, 0.31–0.97; *P* = .04; Fig. 5). No significant differences were evident between the 2 regimens of CCRT with respect to nausea (OR, 0.71; 95% CI, 0.41–1.22; *P* = .22; Fig. 5), vomiting (OR, 1.23; 95% CI, 0.34–4.42; *P* = .75; Fig. 5) or diarrhea (OR, 2.14; 95% CI, 0.71–6.48; *P* = .18; Fig. 5).

### 3.4 Risk of bias

Funnel plots and Harbord tests for all of the indices did not show any evidence of publication bias (all *P* > .05). (See Fig. S1, Fig. S2, Fig. S3, and Fig. S4, Supplemental Digital Content, which show the funnel plots; See Doc S5, Supplemental Digital Content, which presents the details of the Harbord tests).

## 4 Discussion

Cisplatin-based CCRT is the standard regimen used for treatment of LACC according to the National Comprehensive Cancer Network guidelines based on the results of 5 randomized trials.^[2–6]^ However, the optimal regimen using cisplatin in chemotherapy is still unknown. Weekly 40 mg/m^2^ cisplatin and triweekly 75 mg/m^2^ cisplatin are widely used as the most common doses and dosing schedules at present. In a randomized clinical trial by Ryu et al, triweekly cisplatin-based CCRT was associated with better 5-year survival and a lower incidence of hematological toxicity than the conventional weekly cisplatin in patients with LACC.^[16]^ Nevertheless, 2 meta-analyses comparing concurrent weekly cisplatin to triweekly cisplatin-based CCRT for treatment of cervical cancer suggested the superiority of the weekly cisplatin regimen based only on the lower incidence of hematological toxicity.^[9,10]^ These 2 meta-analyses included a small number of randomized controlled trials, which might explain why neither of them analyzed the differences in hemoglobin levels between the 2 groups during treatment.

In this meta-analysis, we found that triweekly cisplatin-based (20 mg/m^2^ for 5 days or 75 mg/m^2^) CCRT was associated with a lower rate of local recurrence and anemia than weekly cisplatin-based (40 mg/m^2^) CCRT in patients with LACC. We observed that patients with a high level of hemoglobin during CCRT might have better local control of LACC based on the results of the triweekly cisplatin treatment group. In a retrospective study by Obermair et al, the authors found that a high level of hemoglobin was an independent predictor of a better prognosis in LACC during CCRT.^[19]^ We also found that anemia was reported to have associations with impaired local control and decreased OS in a large series of patients with cervical carcinoma.^[20,21]^ Therefore, we can propose that a triweekly cisplatin regimen might improve the local control of CCRT for LACC in part by maintaining high hemoglobin levels. Cisplatin, taken as a radiosensitizer, greatly improves the efficiency of radiation. Meanwhile, tumor hypoxia plays an important role in the radiosensitivity of the tumor itself, which also influences the treatment outcomes. Hypoxia can increase spontaneous aggressiveness, tumor angiogenesis, and relative tumor resistance, which can be considered the main reasons underlying resistance to RT by mediating molecular changes related to cellular processes.^[22]^ In addition, tumor vascularity, tumor perfusion, oxygen consumption, and reoxygenation are closely related to the overall oxygenation state of the cancer.^[23]^ Therefore, a falling hemoglobin level may contribute to radiation resistance by increasing the proportion of hypoxic cells in the tumor.

Multiple factors contribute to anemia, including hemorrhage and CCRT. In the studies included in our meta-analysis, the regimen of cisplatin was the only intervention factor. We may make some assumptions regarding the relative high level of hemoglobin in the triweekly cisplatin group. One alternate hypothesis is that cisplatin-based chemotherapy with a triweekly regimen resulted in less damage to erythrocytes compared with leukocytes and platelets. Another possible explanation could be that many ancillary therapies, such as transfusions and the use of erythropoietin, might maintain the level of hemoglobin. However, relevant clarification and analysis were not conducted in these articles. Another reason that might explain the better local control is the improved or sustained high peak blood levels with cisplatin might directly decrease the fraction of hypoxic cells in the tumors, resulting in a decrease in local failure and an eventual survival benefit. The cause for better local control and less anemia in the triweekly cisplatin group and the exact association between the 2 phenomena are still unknown and further relevant studies are warranted.

Additionally, we observed that the incidence of leukopenia and thrombocytopenia were higher in the triweekly cisplatin arm, which is similar to the findings of a previous meta-analysis.^[10]^ Several studies have shown that a higher cumulative dose of cisplatin might lead to a better tumor control to a certain degree.^[24,25]^ However, severe adverse effects, especially regarding the hematologic toxicity, interrupted or postponed the courses in many patients.^[26–28]^ Though our meta-analysis showed that patients in the triweekly cisplatin group suffered more leukopenia and thrombocytopenia, no difference was observed in the courses of chemotherapy between the triweekly regimen group and the weekly group. Maintaining a high level of cisplatin dose with tolerable adverse effects might contribute to the better locoregional control.

Although no significant difference was shown in terms of compliance in our meta-analysis, subgroup analysis was conducted, and interesting results were observed. We found that patients that received triweekly regimen had poorer RT completion (*P* = .05). A strong trend of better compliance was also observed in patients treated with triweekly cisplatin-based CCRT after 2009, while the opposite phenomenon took place up to 2008 (including 2008), though no statistical significance was found. As previously mentioned, in the retrospective study of Einstein in 2006,^[29]^ inpatients receiving the triweekly cisplatin regimen had a longer duration of treatment and more acute adverse events might be observed, as well as an increase in the use of prophylactic medications. Therefore, inpatients might have a higher probability of suspension or termination of treatment. However, there is a trend for outpatients to use weekly cisplatin due to the ease of weekly dosing and the lower cost of outpatient administration. Acute adverse events might be ignored or tolerated while the patient is out of the hospital, resulting in early toxicities being underreported or not well documented in the weekly dosage group. The opposite results observed after 2008 might be the result of a more accurate and individualized RT technology and more effective treatment measures available at the time. In addition, hospitals with comprehensive facilities and improved conditions tend to accommodate more patients regardless of their physical condition. Another interesting result of subgroup analysis demonstrated that patients treated with triweekly cisplatin-based CCRT had a lower rate of RT completion (*P* = .05), although better compliance existed in this group after 2008. We have analyzed this contradictory phenomenon as follows. First, stronger effects of radiation occurred not only in the malignant lesions but also in normal tissue, due to the higher levels of hemoglobin in the triweekly dosage group. The higher probability of damage caused by the side effects of RT might contribute to the delay or suspension of RT. Second, the triweekly group suffered from more hematological toxicity caused by either chemotherapy or RT. Third, 3 studies were used to analyze RT completion, including 1 study in 2008 (155 cases) and 2 studies after 2008 (104 and 60 cases). The different years and the diversity of patient sample size might also account for the lower rate of RT completion, which is contrary to the outcome of better compliance found after 2008.

No significant difference was observed in OS between the 2 regimens, but a trend of better 3-year and 5-year OS was found in the triweekly group. Patients with better local control have been reported to possibly have better survival.^[30,31]^ Therefore, we hypothesized that the higher rate of locoregional control in the triweekly cisplatin regimen may contribute to the improvement of OS. This is in accordance with the conclusion drawn from a meta-analysis comparing the efficacies and acute toxicities in weekly and triweekly cisplatin-based CCRT for advanced head and neck cancer patients, where triweekly cisplatin-based CCRT was recommended.^[30]^ Therefore, we believe that randomized trials with a large sample size are necessary to define the benefits of these 2 regimens.

Several criticisms can be made of our study. First, our meta-analysis included 2 studies where patients received cisplatin every 4 weeks due to the limited number of relevant studies to date. No heterogeneity was observed in the Harbord tests, and the results of our meta-analysis are convincing and trustworthy. Second, 2 studies used cisplatin-based polychemotherapy plus RT in the treatment of LACC. Regimens of triweekly cisplatin in these studies were combined with other chemotherapeutic drugs, such as tirapazamine, 5-FU, hydroxyurea, and paclitaxel. In a meta-analysis by Petrelli et al, the authors found that platinum-based doublet CCRT increased OS and PFS compared to RT plus single platinum-based therapy.^[8]^ However, the data collected from these studies were mainly used for the analysis of acute adverse events. The results of our meta-analysis in terms of acute adverse events showed significant differences only in hematological toxicity, which was consistent with the results of 2 other similar meta-analyses.^[9,10]^

Our study has some limitations. First, due to the diverse methods used to assess treatment outcomes, some favorable characteristics and endpoints such as neurotoxicity and urine tract toxicity, were not analyzed in our study. Additionally, differences with respect to the dose and duration of RT may have also influenced the results. As shown in Table 1, different types of RT technology were used in the studies, but we did not observe a significant difference in dose or duration of RT when analyzing OS and recurrence. No significant heterogeneity of publication was found after Harbord tests were carried out in every analysis. Therefore, we believe the conclusions drawn from our meta-analysis to be accurate to a certain degree. Finally, only published literature was included in this meta-analysis, and the lack of individual patient data prevented us from adjusting for the confounding influences of disease- and patient-related variables on the effect of type of treatment.

## 5 Conclusions

To our knowledge, this is the largest randomized controlled trial review comparing the efficiency and adverse events associated with triweekly and weekly cisplatin-based CCRT in patients with LACC. We found that a lower rate of local relapse and anemia occurred in the triweekly cisplatin group. Therefore, a triweekly cisplatin regimen could be an optimal regimen for CCRT in patients with LACC. The outcome of our meta-analysis could serve as a hypothesis-generating platform for more prospectively randomized phase II and III clinical trials for this patient population in the future.

## Acknowledgments

The authors would like to thank the members of the research group for their useful discussions.

## Author contributions

**Conceptualization:** Shengjun Ji.

**Data curation:** Jiahao Zhu, Qingqing Chen, Qunchao Hu.

**Formal analysis:** Jiahao Zhu, Qingqing Chen, Qunchao Hu.

**Funding acquisition:** Ke Gu.

**Investigation:** Qunchao Hu, Shengjun Ji.

**Methodology:** Zheng Zhang, Dongyan Bian, Qunchao Hu.

**Project administration:** Shengjun Ji, Ke Gu.

**Resources:** Shengjun Ji.

**Software:** Jiahao Zhu.

**Supervision:** Dongyan Bian, Qingqing Chen, Qunchao Hu, Ke Gu.

**Writing – original draft:** Jiahao Zhu.

**Writing – review and editing:** Jiahao Zhu, Zheng Zhang, Dongyan Bian, Shengjun Ji.

Shengjun Ji orcid: 0000-0002-8862-6256.

## Supplementary Material

Supplemental Digital Content

## Supplementary Material

Supplemental Digital Content

## Supplementary Material

Supplemental Digital Content

## Supplementary Material

Supplemental Digital Content

## Supplementary Material

Supplemental Digital Content
