# Supplementary figures and images for: Weekly versus triweekly cisplatin-based concurrent chemoradiotherapy in the treatment of locally advanced cervical carcinoma: An updated meta-analysis based on randomized controlled trials
Source: Medicine (Baltimore). 2020 Jan 3;99(1):e18663. doi: 10.1097/MD.0000000000018663 (PMC6946561; doi:10.1097/MD.0000000000018663)

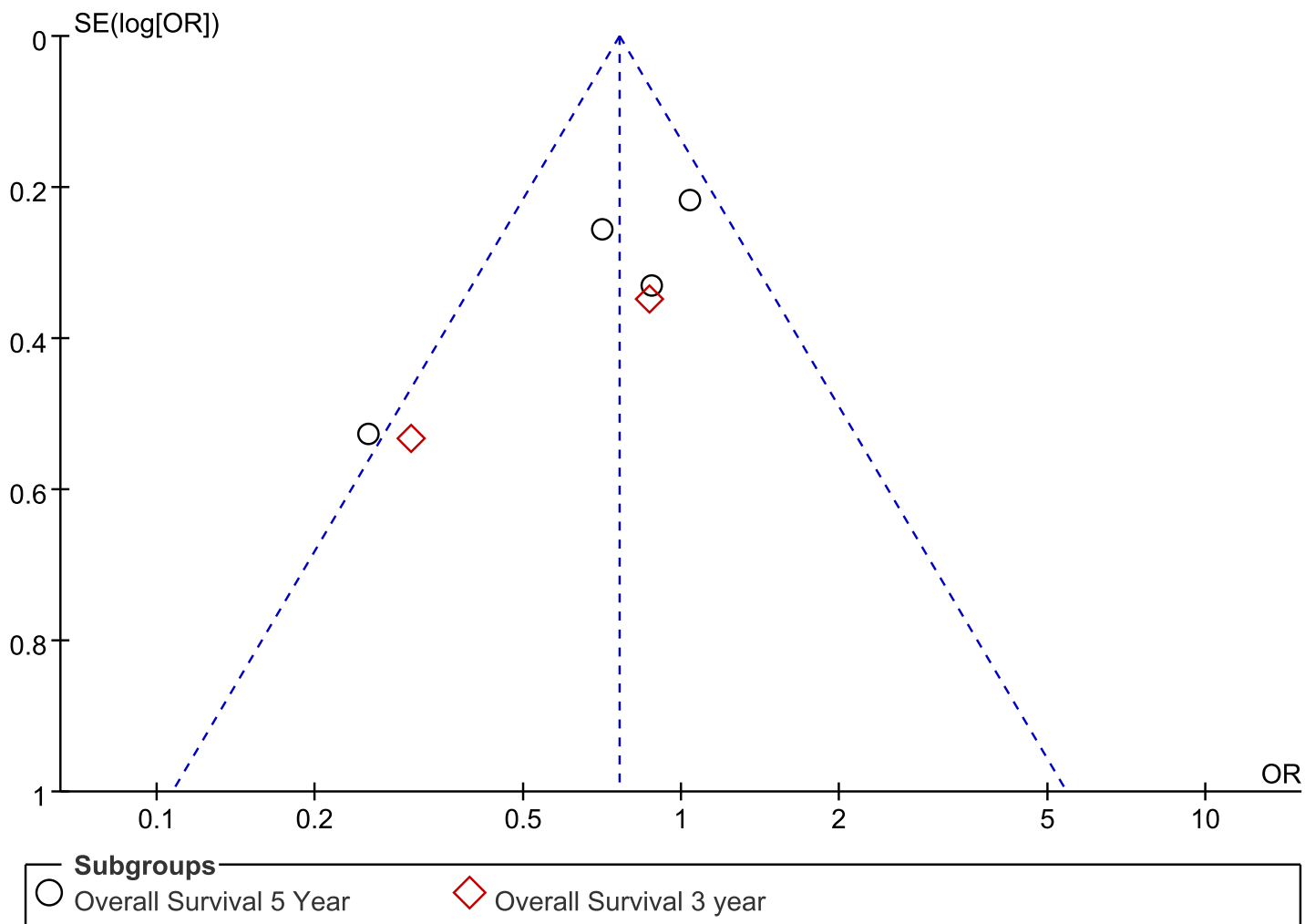

Supplement: Supplemental Digital Content [file medi-99-e18663-s001.pdf]

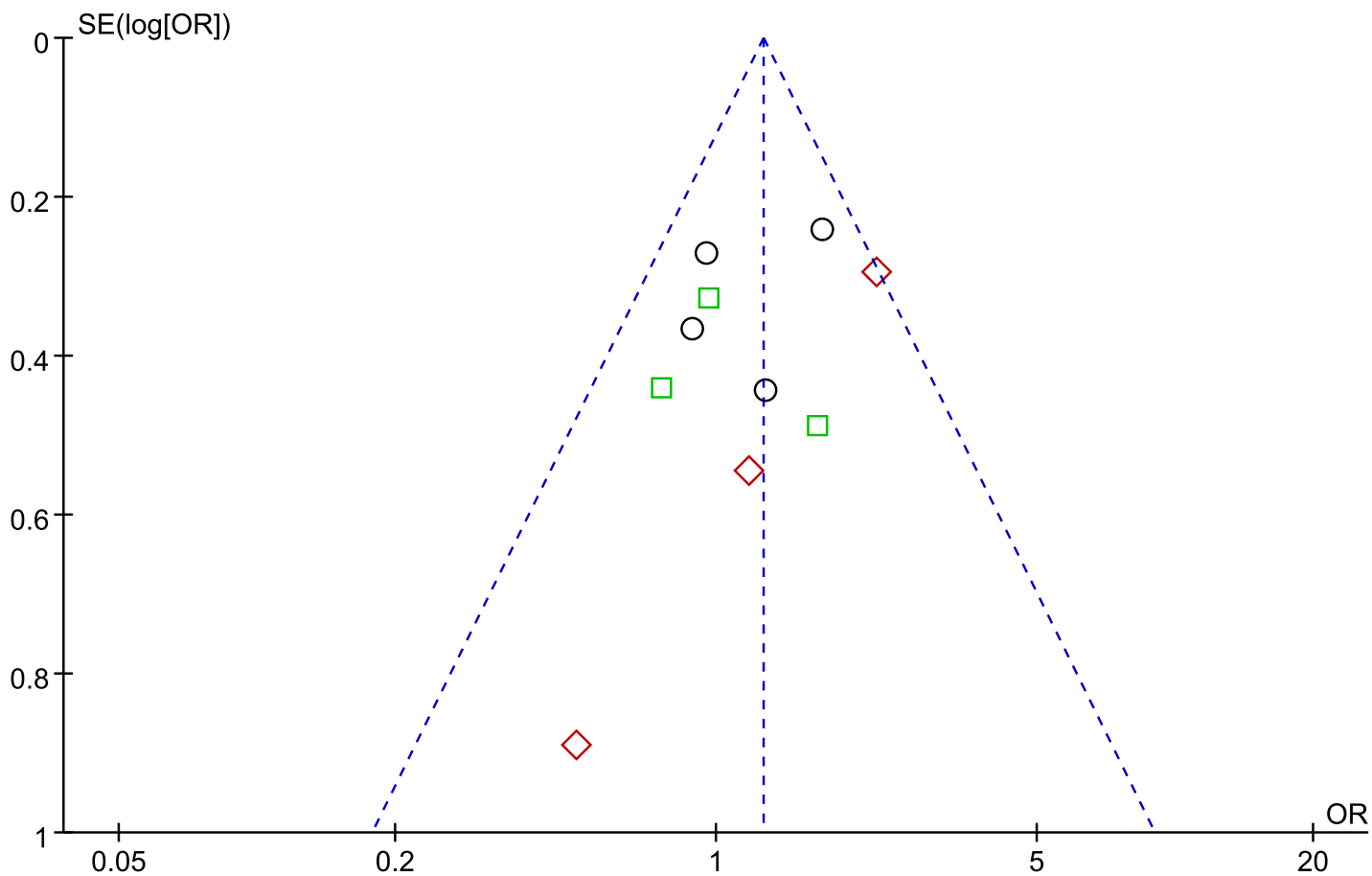

**Subgroups**

○ Recurrence 5 Year      ◇ Recurrence Local 5 Year      □ Recurrence Distance 5 Year

Supplement: Supplemental Digital Content [file medi-99-e18663-s002.pdf]

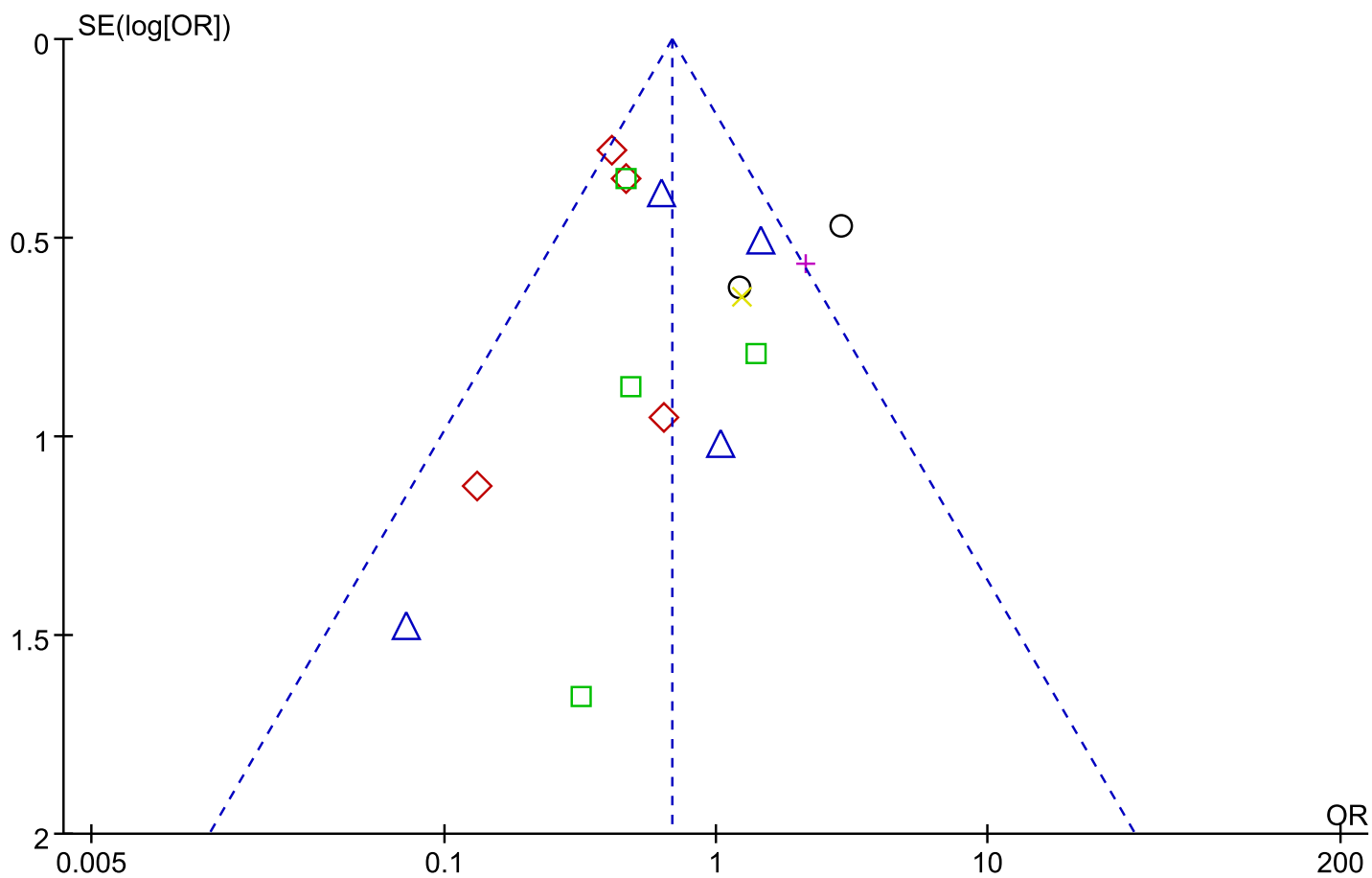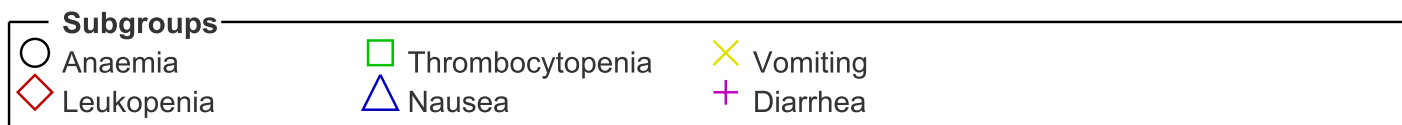

Supplement: Supplemental Digital Content [file medi-99-e18663-s004.pdf]
