# Supplementary material for: Weekly versus triweekly cisplatin-based concurrent chemoradiotherapy in the treatment of locally advanced cervical carcinoma: An updated meta-analysis based on randomized controlled trials
Source: Medicine (Baltimore). 2020 Jan 3;99(1):e18663. doi: 10.1097/MD.0000000000018663 (PMC6946561; doi:10.1097/MD.0000000000018663)
Supplement: Supplemental Digital Content [file medi-99-e18663-s003.pdf]

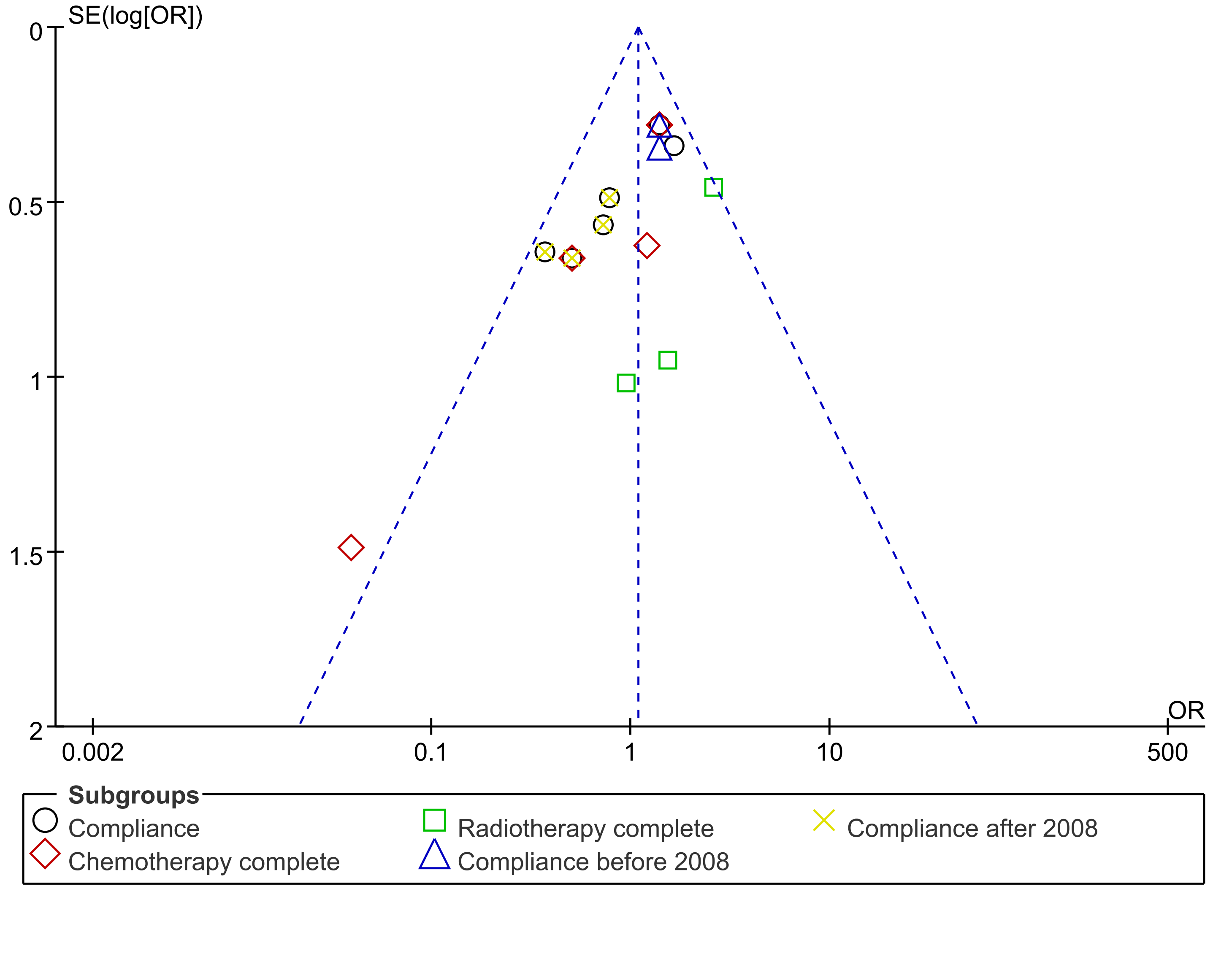
SE(log[OR])
0
0.5
1
1.5
2
OR
0.002
0.1
1
10
500
Subgroups
Compliance
Radiotherapy complete
Compliance after 2008
Chemotherapy complete
Compliance before 2008
