# Supplementary material for: Weekly versus triweekly cisplatin-based concurrent chemoradiotherapy in the treatment of locally advanced cervical carcinoma: An updated meta-analysis based on randomized controlled trials
Source: Medicine (Baltimore). 2020 Jan 3;99(1):e18663. doi: 10.1097/MD.0000000000018663 (PMC6946561; doi:10.1097/MD.0000000000018663)
Supplement: Supplemental Digital Content [file medi-99-e18663-s005.doc]

metabias qwevent qwtotal q3wevent q3wtotal, harbord graph

Note: data input format tcases tnoncases ccases cnoncases assumed.

Note: odds ratios assumed as effect estimate of interest

Harbord's modified test for small-study effects:

Regress Z/sqrt(V) on sqrt(V) where Z is efficient score and V is score variance

1. OS-5 Year

Number of studies = 4 Root MSE = .4642

------------------------------------------------------------------------------

Z/sqrt(V) | Coef. Std. Err. t P>|t| [95% Conf. Interval]

-------------+----------------------------------------------------------------

sqrt(V) | .1895405 .2073746 0.91 0.457 -.7027206 1.081802

bias | -1.283524 1.006884 -1.27 0.330 -5.615796 3.048748

------------------------------------------------------------------------------

Test of H0: no small-study effects P = 0.330

1. OS-3 Year

Number of studies = 2 Root MSE = 0

------------------------------------------------------------------------------

Z/sqrt(V) | Coef. Std. Err. t P>|t| [95% Conf. Interval]

-------------+----------------------------------------------------------------

sqrt(V) | 1.018441 . . . . .

bias | -4.230186 . . . . .

------------------------------------------------------------------------------

Test of H0: no small-study effects P = .

1. Recurrence 5 Year

Number of studies = 4 Root MSE = .844

------------------------------------------------------------------------------

Z/sqrt(V) | Coef. Std. Err. t P>|t| [95% Conf. Interval]

-------------+----------------------------------------------------------------

sqrt(V) | .5226444 .5201005 1.00 0.421 -1.715168 2.760456

bias | -1.347849 1.792938 -0.75 0.531 -9.062236 6.366539

------------------------------------------------------------------------------

Test of H0: no small-study effects P = 0.531

1. Recurrence Local 5 Year

Number of studies = 3 Root MSE = .1436

------------------------------------------------------------------------------

Z/sqrt(V) | Coef. Std. Err. t P>|t| [95% Conf. Interval]

-------------+----------------------------------------------------------------

sqrt(V) | 1.242895 .0822684 15.11 0.042 .1975756 2.288215

bias | -2.148912 .199961 -10.75 0.059 -4.689657 .3918331

------------------------------------------------------------------------------

Test of H0: no small-study effects P = 0.059

1. Recurrence Distance 5 Year

Number of studies = 3 Root MSE = .917

------------------------------------------------------------------------------

Z/sqrt(V) | Coef. Std. Err. t P>|t| [95% Conf. Interval]

-------------+----------------------------------------------------------------

sqrt(V) | -.5607623 1.292816 -0.43 0.739 -16.98755 15.86603

bias | 1.485532 3.29661 0.45 0.730 -40.40187 43.37294

------------------------------------------------------------------------------

Test of H0: no small-study effects P = 0.730

1. Compliance

Number of studies = 6 Root MSE = .4486

------------------------------------------------------------------------------

Z/sqrt(V) | Coef. Std. Err. t P>|t| [95% Conf. Interval]

-------------+----------------------------------------------------------------

sqrt(V) | .1393241 .1128251 1.23 0.284 -.1739286 .4525768

bias | -.6044178 .5007382 -1.21 0.294 -1.99469 .7858543

------------------------------------------------------------------------------

Test of H0: no small-study effects P = 0.294

1. Chemotherapy complete

Number of studies = 4 Root MSE = .4901

------------------------------------------------------------------------------

Z/sqrt(V) | Coef. Std. Err. t P>|t| [95% Conf. Interval]

-------------+----------------------------------------------------------------

sqrt(V) | .2137838 .1579847 1.35 0.309 -.4659695 .8935372

bias | -.9563136 .6322107 -1.51 0.270 -3.676497 1.763869

------------------------------------------------------------------------------

Test of H0: no small-study effects P = 0.270

1. Radiotherapy complete

Number of studies = 3 Root MSE = .3418

------------------------------------------------------------------------------

Z/sqrt(V) | Coef. Std. Err. t P>|t| [95% Conf. Interval]

-------------+----------------------------------------------------------------

sqrt(V) | .3262367 .3183006 1.02 0.492 -3.718156 4.370629

bias | -.8951012 1.12819 -0.79 0.573 -15.23011 13.43991

------------------------------------------------------------------------------

Test of H0: no small-study effects P = 0.573

1. Compliance before 2008

Number of studies = 2 Root MSE = 0

------------------------------------------------------------------------------

Z/sqrt(V) | Coef. Std. Err. t P>|t| [95% Conf. Interval]

-------------+----------------------------------------------------------------

sqrt(V) | -.0226588 . . . . .

bias | .5126588 . . . . .

------------------------------------------------------------------------------

Test of H0: no small-study effects P = .

1. Compliance after 2008

Number of studies = 4 Root MSE = .4122

------------------------------------------------------------------------------

Z/sqrt(V) | Coef. Std. Err. t P>|t| [95% Conf. Interval]

-------------+----------------------------------------------------------------

sqrt(V) | .2049567 .186903 1.10 0.387 -.599222 1.009135

bias | -.6482215 .9192835 -0.71 0.554 -4.603579 3.307136

------------------------------------------------------------------------------

Test of H0: no small-study effects P = 0.554

1. Anaemia

Number of studies = 2 Root MSE = 0

------------------------------------------------------------------------------

Z/sqrt(V) | Coef. Std. Err. t P>|t| [95% Conf. Interval]

-------------+----------------------------------------------------------------

sqrt(V) | 1.654834 . . . . .

bias | -2.473366 . . . . .

------------------------------------------------------------------------------

Test of H0: no small-study effects P = .

1. Leukopenia

Number of studies = 4 Root MSE = .7293

------------------------------------------------------------------------------

Z/sqrt(V) | Coef. Std. Err. t P>|t| [95% Conf. Interval]

-------------+----------------------------------------------------------------

sqrt(V) | -.4936023 .3119115 -1.58 0.254 -1.835649 .8484445

bias | -.442583 .806184 -0.55 0.638 -3.911313 3.026147

------------------------------------------------------------------------------

Test of H0: no small-study effects P = 0.638

1. Thrombocytopenia

Number of studies = 4 Root MSE = .8965

------------------------------------------------------------------------------

Z/sqrt(V) | Coef. Std. Err. t P>|t| [95% Conf. Interval]

-------------+----------------------------------------------------------------

sqrt(V) | -.3411641 .4675643 -0.73 0.541 -2.352931 1.670603

bias | -.2143945 .8422281 -0.25 0.823 -3.838209 3.40942

------------------------------------------------------------------------------

Test of H0: no small-study effects P = 0.823

1. Nausea

Number of studies = 4 Root MSE = 1.594

------------------------------------------------------------------------------

Z/sqrt(V) | Coef. Std. Err. t P>|t| [95% Conf. Interval]

-------------+----------------------------------------------------------------

sqrt(V) | .2252843 1.206319 0.19 0.869 -4.965086 5.415654

bias | -1.101855 2.221329 -0.50 0.669 -10.65946 8.455752

------------------------------------------------------------------------------

Test of H0: no small-study effects P = 0.669
